# High-Throughput White Blood Cell (Leukocyte) Enrichment from Whole Blood Using Hydrodynamic and Inertial Forces

**DOI:** 10.3390/mi11030275

**Published:** 2020-03-06

**Authors:** Batzorig Lombodorj, Horas Cendana Tseng, Hwan-You Chang, Yen-Wen Lu, Namnan Tumurpurev, Chun-Wei Lee, Batdemberel Ganbat, Ren-Guei Wu, Fan-Gang Tseng

**Affiliations:** 1 School of Information and Communication Technology, Mongolian University of Science and Technology, Ulaanbaatar 14191, Mongolia; batzorig.l2007@gmail.com; 2 Department of Engineering and System Science, Frontier Research Center on Fundamental and Applied Sciences of Matters, National Tsing Hua University, Hsinchu 30013, Taiwan; Horas.tseng@gmail.com (H.C.T.); waynelee19850217@hotmail.com (C.-W.L.); 3 Department of Life Science, National Tsing Hua University, Hsinchu 30013, Taiwan; hychang@life.nthu.edu.tw; 4 Department of Biomechatronics Engineering, National Taiwan University, Taipei 10617, Taiwan; 5 Department of Mechanical Engineering, Mongolian University of Science and Technology, Ulaanbaatar 14191, Mongolia; namnan@must.edu.mn; 6 Department of Physics, Mongolian University of Science and Technology, Ulaanbaatar 14191, Mongolia; gdembee@must.edu.mn; 7 Research Center for Applied Sciences, Academia Sinica, Taipei 11529, Taiwan

**Keywords:** blood separation, microfluidics

## Abstract

A microfluidic chip, which can separate and enrich leukocytes from whole blood, is proposed. The chip has 10 switchback curve channels, which are connected by straight channels. The straight channels are designed to permit the inertial migration effect and to concentrate the blood cells, while the curve channels allow the Dean flow to further classify the blood cells based on the cell sizes. Hydrodynamic suction is also utilized to remove smaller blood cells (e.g., red blood cell (RBC)) in the curve channels for higher separation purity. By employing the inertial migration, Dean flow force, and hydrodynamic suction in a continuous flow system, our chip successfully separates large white blood cells (WBCs) from the whole blood with the processing rates as high as 1 × 10^8^ cells/sec at a high recovery rate at 93.2% and very few RBCs (~0.1%).

## 1. Introduction

Blood is an important compartment of the body; it circulates in the body and helps to keep the functions of life. Since blood may contain disease information, it is collected and stored for medical treatment when needed, with this information widely applied in disease detection and medical treatment [1]. As blood is composed of various types of cells, including erythrocytes (i.e., red blood cells (RBCs)), leukocytes (i.e., white blood cells (WBCs)), and thrombocytes (i.e., platelets (PLTs)), the capability to effectively separate blood cells can provide us with vital information for diagnosis of disease immune deficiency and genetic abnormalities [2,3,4].

In blood cell separation, size-based filtration using porous membranes has been a popular approach, although it may suffer from clogging, high flow stress and cell damage. On the other hand, microfluidics have been demonstrated to separate particles or cells, including pinched flow fractionation (PFF) [5,6,7,8,9], cross-flow filtration [10,11,12,13,14], microfluidic disk [15], laminar vortices [16,17,18], centrifugation/inertial focusing [19,20,21], and Dean flow [22,23,24,25]. Cell separation using microfluidics on a microchip provides advantages over conventional methods by scaling down the instrumentation size, simplifying the operation procedures for automation, as well as enabling the downstream integration for cellular isolation, analysis, and processing.

Recently, particle separation in microfluidics using inertial migration has received much attention because of its possibility in high throughput applications with simple designs [26,27,28,29,30,31]. The particles/cells flow in rectangular channels are focused near the cross-sectional corners of the channels due to the wall-crowding effect, whereby the wall repulsion forces push the particle away from the wall while the shear-gradient force draws the particle towards the wall. Hence, the balance between these two oppositely directed forces induce an equilibrium force at certain equilibrium positions [32]. 

Although the inertial migration technique permits high-throughput separation, higher separation efficiency is still desirable when it applies to separate biological cells (e.g., blood cells or rare cells) that usually have a large variety of sizes, shapes and rigidities for clinical applications. Herein, we propose to enhance the separation purity and efficiency of the inertial migration by adding (1) curve channels for Dean flow, (2) trench structures to induce additional secondary flows, as well as (3) hydrodynamic suction to improve the separation purity. Our microchip is designed to possess the advantage of the inertial migration with Dean flow and hydrodynamic suction; it has been created and successfully shows the RBC separation from whole blood with the WBC enrichment in a continuous flow system.

## 2. Device Design and Working Principle 

**Figure 1:**
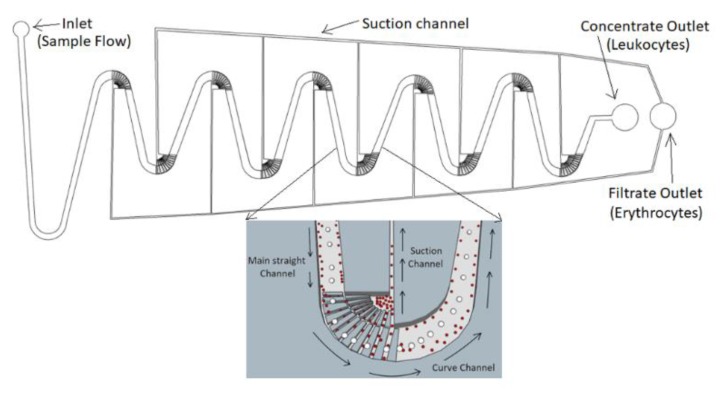
Our separation chip has three types of channel: (i) a main straight channel, (ii) curve channels, and (iii) suction channels. The main straight channel has the inertial migration to move the cells into different equilibrium regions. The curve channels (with the close-up view in the inset) push the white blood cells (WBCs) toward the outer part while the red blood cells (RBCs) move toward the inner part. The suction channel allows the RBCs to be sucked away. Note: the main dimensions of the microchannels used in this work are: W = 200 μm, H = 58 or 85 μm, radius of curvature = 100 μm, and W (suction) = 7 μm.

Figure 1 depicts the design of our separation chip with three types of channel: (i) a main straight channel, (ii) curve channels, and (iii) suction channels. Each type of the channel plays different roles to separate the cells. The whole separation chip consists of ten switchback curve channels connected by the main straight channel. The sample flow is injected from the inlet, passes through the main channel, then through the curve channel, which has the sample flow eventually injecting higher concentrations into the “concentrate outlet”. Meanwhile, each curve channel has one suction channel at the inner curve with all suction channels being connected to draw the smaller blood cells then collected into the filtrate outlet. 

(1) Main Straight Channel for Inertial Migration Effect

**Figure 2:**
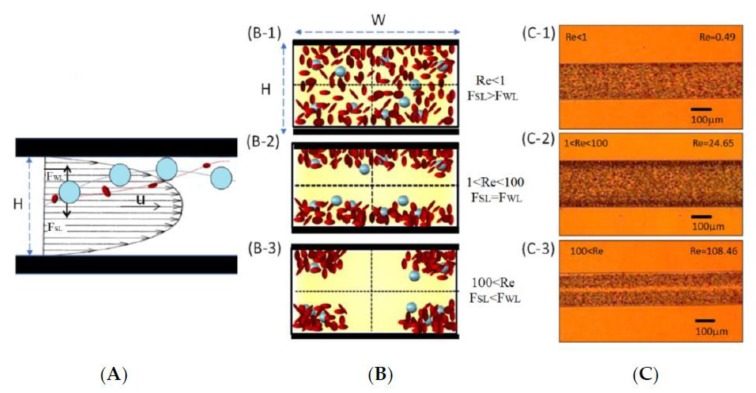
(**A**) A particle/cell experiences the wall-induced lift force (*F_WL_*) and shear-gradient lift force (*F_SL_*) inside a microchannel with a WxH cross section. The small particles/cells (e.g., RBC) tend to move toward the corners of the microchannel as the lift forces are larger than the drag forces. The large particles/cells (e.g., WBC) tend to move toward the outer region of the microchannels. (**B**) Due to the inertial migration effect, the cells with different sizes move into different cross-sectional regions in the microchannel. Based on the relationship between F_D_ and F_L_, the cells are: (B-1) uniformly distributed, when *F_SL_* > *F_WL_*, or Re < 1; (B-2) close to the top and bottom walls of the microchannels, when *F_SL_* = *F_WL_*, or 100 > Re > 1; and (B-3) around the corners of the microchannels when *F_SL_* < *F_WL_*, Re > 100. (**C**) The microscopy views of the whole bloods flowing in the microchannel with (C-1) blood cells distribute uniformly at 5 μL/sec or Re < 1; (C-2) blood cells concentrate close to the top, bottom and sidewall surfaces at 250 μL/sec, or 100 > Re > 1; (C-3) blood cells concentrate around the corner of the microchannel at 1.1 mL/sec, or Re > 100.

The main straight channel with rectangular cross sections is located in the entrance, and connects the curve channels. As Poiseuille flow naturally presents in the microchannels, particles/cells usually are acted upon by two fluid dynamic effects—wall-induced lift forces and shear-gradient lift forces—for their discrete equilibrium positions [33,34]. The wall-induced lift forces push the particles/cells away from the walls, while the shear-gradient lift forces push the particles/cells towards the walls [35]. These two effects result the particles/cells with different sizes in equilibrium positions between the channel walls and centerlines, as shown in Figure 2A.

Furthermore, the wall-induced lift force ($F_{WL}$) encountered by the particles/cells can be expressed by Stoke’s law.

(1) $$F_{WL}=3\pi \mu a_{p}U_{L}$$

where *μ* is the fluid viscosity, $a_{p}$ is the particle diameter and $U_{L}$ is the minimum fluid velocity to induce the particles moving toward the channel walls.

The shear-gradient lift force ($F_{SL}$) on particles/cells derived from a shear-gradient flow field can be expressed as:

(2) $$F_{SL}=\frac{2\rho U_{f}^{2}a_{p}^{4}}{D_{h}^{2}}$$

(3) $$D_{h}=\frac{4A}{P}=\frac{2WH}{\left(W+H\right)}$$

where ρ is the fluid density, $U_{f}$ is the flow average velocity in a microchannel and $D_{h}$ is the hydraulic diameter for the microchannel. 

Based upon the above expressions, the values of these two forces of $F_{WL}$ and $F_{SL}$ depend on the particle/cell sizes ($a_{p}$). By varying the values of these two forces, $F_{WL}$ and $F_{SL}$ differential migration of particles/cells based on their sizes can be utilized for separation. Figure 2B depicts the three possible situations for $F_{WL}>F_{SL}$, $F_{WL}=F_{SL}$, and $F_{WL}<F_{SL}$ Moreover, when the cells reach the equilibrium at $F_{WL}=F_{SL}$, the terminal velocity *U_L_* of the cells can be expressed as:

(4) $$U_{L}=\frac{F_{SL}}{3\pi \mu a_{p}}=\frac{2\rho U_{f}^{2}a_{p}^{3}}{3\pi \mu {\left(\frac{2WH}{W+H}\right)}^{2}}$$

(5) L=Umax(12W)UL=3πW2Re(Dhap)3

where $U_{max}$ is the maximum velocity in a parabolic flow field, and it is commonly assumed as $U_{max}\sim 2U_{f}$. *L* is the channel length required for cells to reach lateral equilibrium position in rectangular channels. 

For better separation, a microchannel, whose width is larger than the height (e.g., aspect ratio = (height/width) <1), is devised, leading large lift forces to move cells laterally toward the channel sidewalls for the next stage of the cell separation in the curve channels. The larger lift forces moreover can reduce the channel length required for cell migration and create better cell separation. Thus, our main straight channel is designed by considering a proper Re numbers (e.g., 100 > Re > 1), aspect ratio < 1 and $F_{WL}=F_{SL}$ so the inertial migration can effectively separate RBCs/WBCs within short distances and prepare for the separation in the next curve channels.

**Figure 3:**
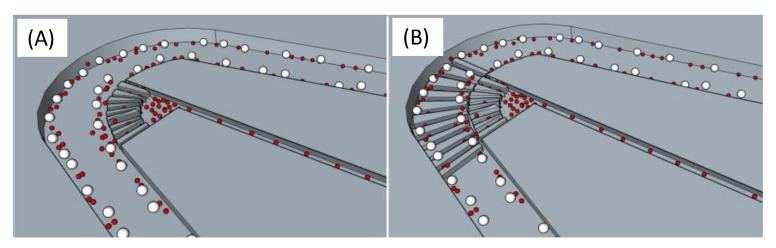
When the blood cells move along the curve channels, some of the smaller cells are sucked into the suction channels. Two designs—(**A**) without obstacle and (**B**) with an array of obstacles—are tested to compare the suction efficiency in the curve channels.

A series of the flow rates are tested at 5 μL/sec, 250 μL/sec, and 1.1 mL/sec to evaluate the cell separation performance with the whole blood using the inertial migration effect in the main straight channels. The channel width and height are 200 and 58 μm. Figure 2C demonstrates the results of this preliminary study. It is found that the blood cells distribute quite uniformly at 5 μL/sec (or Re < 1) for Figure 2C(C-1). The blood cells begin to concentrate around the top and bottom walls when the flow rate increases (e.g., 250 μL/sec or 100 > Re > 1 for Figure 2C(C-2). Once the flow rate further increases, the blood cells move and concentrate around the corners of the microchannel (e.g., 1.1 mL/sec or Re > 100) for Figure 2C(C-3). The inertial migration effect is confirmed for our initial RBC/WBC separation. 

(2) Curve Channel for Dean Flow 

Since the inertial migration could provide a RBCs/WBCs separation efficiency at ~89.8% [36,37] in the straight rectangular channel, a secondary flow by using Dean flow is introduced to further enhance the separation efficiency. A series of curve channels are added to provide Dean flow, which can aid lateral particle/cell migration. Depending on particle/cell size, these secondary Dean flows tend to entrain particles in one of the two vortices formed and force them to follow fluid movement within the vortices. A dimensionless parameter, Dean number (De), is usually defined as:

(6) De=ReDhR

where R is the curvature radius of the curve channel. When De is large enough, the secondary flow forms Dean vortices, vertical to the flow inside the channel. Due to these Dean flows, the drag force exerted on a particle. Larger cells (e.g., WBC) experience larger forces and move toward the outer part of the curve channels; the smaller cells (e.g., RBC) have smaller forces and remain in the inner part. 

(3) Suction Channel for Hydrodynamic Suction

When the blood cells move along the curve channels, some of the smaller cells are sucked into the suction channels. To enhance the separation performance in the suction channels, an array of obstacle structures, or trench structures, are induced in the curve channel to create additional secondary flows so better separation can be obtained before the suction. To compare their effects in the cell separation efficiency, two designs—(A) without obstacle and (B) with an array of obstacles—in the curve channels, as illustrated in Figure 3, are tested. In Design (A), although the Dean flow moves the RBCs toward the inner part of the channels, these RBCs mostly keep moving along the curve channel, together with WBCs. Contrarily, in Design (B), some of the RBCs falls inside the trenches (with the height at 15 μm) and are guided into the suction channels, while the WBCs roll over the obstacles and keep moving along the main channel. As a result, some of the RBCs are removed from the sample flow, while the rest of the blood cells stay downstream for further separation. The RBCs can be collected from the suction channels and the recovery rate can be enhanced.

Finally, our device has Dean number (De) approximately set at 11.33, as the radius of the curvature is 200 μm. This Dean number is in the range for Dean flow effect in the curve channel with the geometric parameters of the straight and the curve channels, as well as the flow rate. 

## 3. Material and Methods

### 3.1. Fabrication of Our Separation Microchip

**Figure 4:**
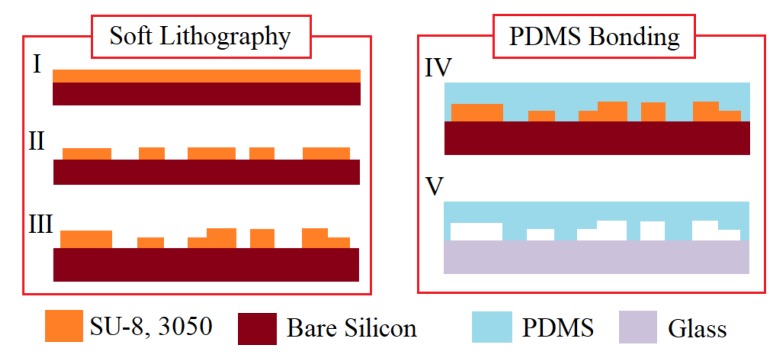
Fabrication process of the blood cells separation device. I-III are the steps for standard lithography with the negative photoresist SU8. IV–V are the steps for soft lithography with polydimethylsiloxane (PDMS) and then bonding the device on a glass substrate after plasma treatment. Note: (I–III), the SU8 process follows the standard procedure, provided from official data sheet. A two layers of SU8 are made: a first SU8 layer with a 20–50 μm thickness is spun-coated at 1500 rpm, exposed and baked; a second SU8 layer is then coated upon, exposed and baked with similar processing parameters.

To create the main straight channels and curve channels, the molds were first fabricated by patterning two layers of SU-8 3050 (Nippon Kayaku, Tokyo, Japan) negative photoresists on silicon wafers. Polydimethylsiloxane (PDMS, Sylgard 184, Dow Corning, Midland, MI, USA) prepolymer was mixed in 10:1 (*w*/*w*) ratio then poured onto the molds for curing for 2 h at 85 °C. The PDMS structures were released at room temperature to reduce thermal stresses. Finally, the holes for inlets and outlets were punched with the PDMS molds irreversibly bonded to microscopic glass slides by briefly exposing them to an oxygen plasma. The devices were left at room temperature for eight hours before usage to avoid any further deformation. The whole process flow was illustrated in Figure 4. 

### 3.2. Sample Preparation

Human whole blood, drawn from donors, was mixed with EDTA (Trtriplex^®^, Merck, Kennersburg, NJ, USA) as anticoagulant; the ratio of EDTA to blood is 10:1. The cell concentration was measured at 4.3 × 10^8^ cells/mL using cell counter plates. 

### 3.3. Experimental Setup

The sample flow of the whole blood was introduced into our separation chip. A syringe connected to a device with 1/16″ peek tubing and fittings (Upchurch Scientific, Oak Harbor, WA, USA) provided input flow using a syringe pump (KD Scientific, Holliston, MA, USA). Prior to the experiment, 95% alcohol was injected into the microchannel for pre-treatment with the sample solution injected. The flow behavior was first visualized using an inverted epi-fluorescence microscope (IX71, Olympus Inc., Tokyo, Japan) equipped with a 12-bit high-speed charge-coupled device (CCD) camera. To image cross-sectional flow, confocal microscopy was performed using Zeiss LSM710 LIVE Duo Confocal Microscope with the aforementioned experimental set-up. Cross-sectional images were taken at each micro channel main and section. The images were acquired at resolution of 1024 × 768 pixels. 

### 3.4. Recovery Rate

Recovery rate is one of the most common parameters to quantify the separation efficiency. The recovery rate is defined as the ratio of the number of the cells retrieved from the target outlet to the number of the cells introduced into the inlet.

(7) $$\mathrm{Recovery}\;\mathrm{rate}=\;\frac{{\left(N_{\mathrm{target}}\right)}_{\mathrm{Outlet}}}{{\left(N_{\mathrm{target}}\right)}_{\mathrm{Inlet}}}\times 100\%$$

where $N_{\mathrm{target}}$ was the number of the target cells (e.g., cells of the interests, or WBC in this work).

## 4. Results and Discussion

### 4.1. Inertial Migration Effect 

To investigate the relationship between flow velocity and channel length required for cells totally migrating toward the side of the wall, Reynolds number should be greater than one (1) to ensure the occurrence of this hydrodynamic phenomenon. 

**Figure 5:**
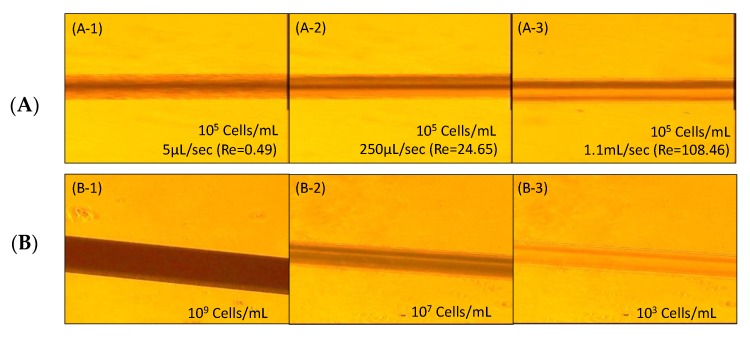
Inertial migration effect in whole blood separation (**A**) with different flow rate, and (**B**) with different blood cells concentration.

Experimental evidence from Figure 5A(A-1–A-3) show the blood separation results when the sample solution is injected at 5 μL/sec, 250 μL/sec and 1.1 mL/sec flow rates (Q_i_). Lateral movements of the cells become obvious, as the flow rates increase. In other words, the lift forces are larger than the drag forces (F_L_ > F_D_) to push the cells moving laterally toward the channel walls at high flow rate, or Re > 100, as shown in Figure 5A(A-3). On the other hand, the lift forces are smaller (F_L_ < F_D_) at low flow rate, or Re < 1

Meanwhile, to identify the effect of the cell concentration, various concentrations at 10^9^ cells/mL, 10^8^ cells/mL and 10^3^ cells/mL are tested, with the results shown in Figure 5B. At cell concentration of 10^8^ cells/mL and flow rate 250 μL/sec, the device provides a separation condition with most of the cells accumulated close to the upper and lower channel walls. It is noted that channel irregularities from the fabrication and unexpected changes in boundary conditions that sometimes create flow deformations or uneven cell distribution, as seen in Figure 5A(A-2,A-3),B(B-2).

### 4.2. Separation Efficiency with Hydrodynamic Suction 

To realize the hydrodynamic suction effects and the separation efficiencies, two different chip designs (e.g., Chip 1 and Chip 2) with a total of 48 chips were tested. Their critical dimensions, including channel width, height, and length were listed in Table 1. Both chip designs were utilized in different flow rate conditions (5 µL/sec, 20 µL/sec, 250 µL/sec and 1.1 mL/sec for Chip 1; 5 µL/sec, 20 µL/sec, 100 µL/sec and 1.1 mL/sec for Chip 2). The optimal flow rates (based on their separation distribution in the main straight channels by visual examinations) were found at 250 µL/sec for Chip 1 and 100 µL/sec for Chip 2. 

**Figure 6:**
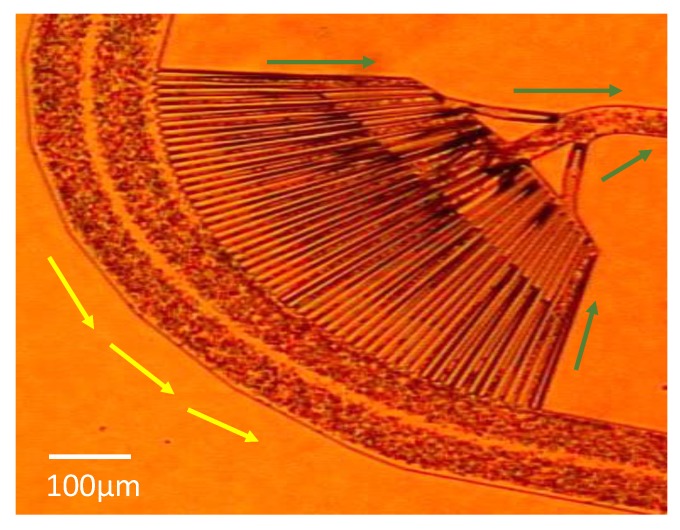
Blood cell separation in the curve channel. Once the sample flow moves along the curve channel, small cells (e.g., RBC) move along the suction channels (green arrow), while larger cells (e.g., WBC and others) move long the curve channels (yellow arrow). Q_i_ = 100 µL/sec; Q_s_ = 20 µL/sec. Chip 1 is used here as an example.

The blood cell separation in the curve channel was examined under an optical microscope. Figure 6 showed the image taken during a test where RBCs and WBCs flow into a curve channel using Chip 1. When Re > 100, the blood cells in the main straight channel would be moving and focused near the corners in the microchannels due to the inertial migration effect. This inertial migration effect increases the number of red blood cells that enter the suction channel, and enhances the cell separation. The larger cells (e.g., WBC and others) move long the curve channels (as shown in the yellow arrows), while a portion of the smaller cells (e.g., RBC) move into the suction channels (as shown by the green arrows).

**Figure 7:**
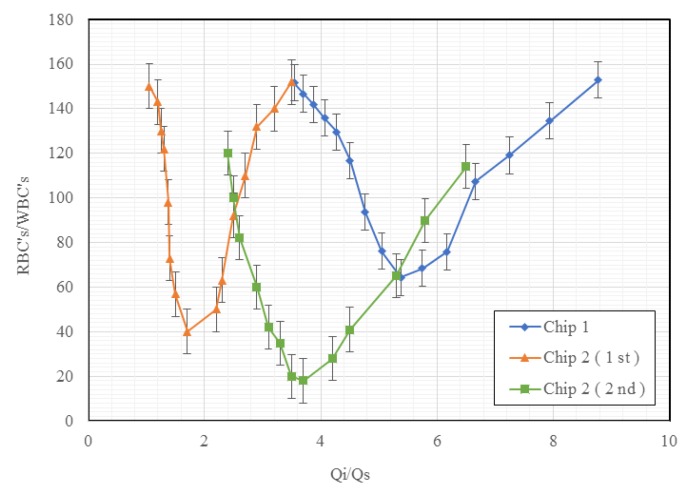
Separation efficiencies to the ratio of injection (Qi) and suction (Qs) flow rates for Chip 1 and Chip 2. (Note: Chip 1 has no obstacle in the curve channels; Chip 2 has obstacles in the curve channels). (Note: Chip 2 (1st and 2nd) has two optimum, as the conditions of the blood samples vary between the 1st and 2nd tests.).

Furthermore, different suction flow rates (Q_s_) were tested to evaluate the separation efficiencies, as depicted in Figure 7. Three experiments were conducted. The ratio of RBCs/WBCs was used as the indicator for separation efficiency, since a low value of RBCs/WBCs represented fewer RBCs and more WBCs in the outlet. A local optimal separation efficiency was found at Q_i_/Q_s_ = 5.4 for Chip 1, and Q_i_/Q_s_ = 1.7 for Chip 2 (the 1st test), and Q_i_/Q_s_ = 3.7 for Chip 2 (the 2nd tests). In other words, when the suction flow rates were too high or too low, the separation efficiencies may not be better than the one with the optimal suction flow rates. This could be because only a few RBCs were removed from the curve channels into the suction channels when Q_s_ was low. Contrarily, although more RBCs were moved into the suction channels, some of the WBCs in the sample flows also were removed into the suction channels when Q_s_ was high, leading the decrease of the separation efficiencies. As the result, the optimal suction flow was found at 37 µL/sec for Chip 1, and at 58 or 27 µL/sec for Chip 2. The separation efficiencies (or RBCs/WBCs) were about 64 for Chip 1, and 40 or 18 for Chip 2. 

### 4.3. Overall Separation Efficiency

**Figure 8:**
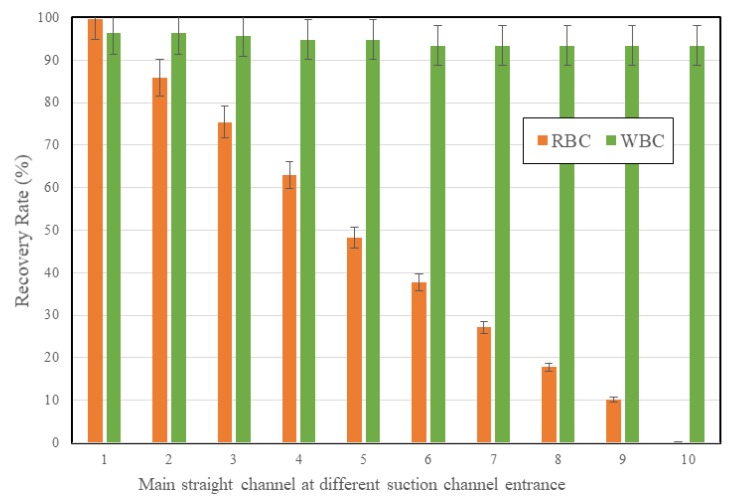
The recovery rate of the RBCs and WBCs in the sample flow after the curve/suction channels—from the first to the tenth curve channels.

The separation performance of the chip was then examined. Figure 8 showed the recovery rates of RBCs and WBCs when the sample flow passed through each curve channel—from the first curve channel to the tenth curve channel, using Chip 2. Passing through the first curve channel, the sample flow had 99.7% of RBCs. It decreased to 85.8% of RBCs when it passed through the second curve channels, or 14.2% of RBCs were removed through the suction channel. It further decreased to 75.4%, or 24.6% of RBCs were removed after the third curve channel. It continued to decrease to 10.2% after the ninth curve channel, and to 0.1% after the tenth curve channel. Therefore, almost all the RBCs in the sample flow were removed through the suction channels after they passed through 10 curve channels. 

On the other hand, the sample flow had 96.2% of WBCs remaining after the first curve channel. The percentage decreased a little bit after every curve channel, and it decreased to 93.2% of WBCs still remaining after the tenth curve channel. The results show a very high recovery rate for WBCs, while very few RBCs were found after the separation chip.

To compare the separation performance, Table 2 exemplifies some of existing inertial and other cell/particle separation techniques in microfluidics. In particular for the separation of biological cells, Y.-Y. Chiu et al. demonstrate hydrodynamic-focusing cross-flow filters in a microfluidic chip to separate PC3 cells from the sample flow (PC3 cells mixed in whole bloods) with a ~94% recovery rate, but at 40 μL/min (or cell processing rate at 4 × 10^6^ cells/sec) [14]. Another research works for cell separation present with a high throughput with processing rates at 7.5 × 10^6^ cells/sec for larger cancer cells spiked in blood from the smaller blood cells [16]. S.Y. Choi and J.K Park report a hydrophoretic filtration device using slanted obstacles to separate WBCs and RBCs from the whole blood with a purity of 76.8% [19]. The flow rate is 1 μL/sec (or cell processing rate at approximate 1 × 10^8^ cells/sec), with over one hour of operation with a recovery rate at 30%. Comparatively, our chip shows great improvement in purity and recovery rate at a high processing rate, or higher throughput in biological cell separation.

As inertial microfluidics (i.e., inertial migration and Dean flow) is a fast-growing field, many related non-linear fluid physics and basic directions are still uncharted. For instance, some analytical models have been presented for straight and curve microchannels for particle/cell separation, but our devices must be re-designed and fabricated for the separations whenever the threshold sizes of the particles/cells change. The flow conditions of the injection and suction flow rates must be experimentally optimized. This is an inherent limitation of our passive inertial microfluidic devices. Also, the physics of how the secondary flow and cell trapping in an obstacle array are created and affected by obstacle arrangement and geometrical parameters is complicated and yet to be understood. Further optimization can be also experimentally conducted for even higher throughput or separation efficiency on these related design variables. Additionally, the interaction of Dean drag and lift forces within the microchannel and related flow condition and channel geometry require diligent experiments. Channel irregularities from the fabrication and unexpected changes in boundary conditions that create flow deformations or uneven cell distribution offer a vast number of other aspects to be studied. 

## 5. Conclusions

We have demonstrated a microfluidic device, which can remove most of the RBCs from whole blood by employing the inertial migration effect, Dean flow, and hydrodynamic suction. An array of obstacles was created in the curve channels, which redirected the RBCs to move into the suction channels. A 93.2% recovery rate of WBCs and very few RBCs in the sample flow were obtained in a high operation flow rate (e.g., 250 μL/sec). Our separation technique, therefore, was advantageous for continuous processing, while reducing the clogging problem—a problem commonly seen in particle separation using a microchannel. Additionally, it provided advantages over conventional methods by scaling down the instrumentation size, simplifying the operation procedures for automation, as well as enabling the downstream integration for cellular isolation, analysis, and processing. It is expected that the results from this work will benefit a wide range of analytical, environmental, biological, and manufacturing applications that require rapid, low-cost, continuous filtration, and/or separation of microscale objects.

## Figures and Tables

**Table 1:** Parameters used for blood cell separation using Chip 1 and Chip 2.

	Microchannel Dimension (µm)	Obstacle in Curve Channel	Sample Solution (Whole Blood)	Flow Rate (µL/sec)
Chip 1	W × H = 200 × 58 R = 200	NO (shown in Figure 3A)	4.3 × 10^8^ cells/mL	5, 20, 250, 1100
Chip 2	W × H = 200 × 85 R = 100	YES (shown in Figure 3B)	4.3 × 10^9^ cells/mL	5, 20, 100, 1100

**Table 2:** Comparison of some existing microfluidic techniques for particle/cell separation.

Channel Type	Mechanism	Particles/Cell Size (D = diameter in μm)	Channel Dimension (μm) Width (W) × Height (H)	Flow Rate (or Throughput)	Recovery Rate, or Capture Efficiency (η)	Ref
Channel with filters	Hydrodynamic focusing, filtration	PC3 cells in whole bloods.	W = 100, H = 30 W_gap_ = 4 (filter)	10 μL/min	94.5% (PC3 cells)	[14]
Trapping reservoir	Inertial/ Trapping by vortices	MCF7 cells; HeLa cells	W = 50 H = 70 m	7.5 × 10^6 ^ cells/sec	η = 10% (HeLa) η = 23% (MCF7)	[16]
Asymmetric serpentine	Differential inertial focusing	Polystyrene beads (D = 3.1, 9.0)	W = 100~650 H = 50	~1.0 mL/min	~56% (3.1 μm particle, two tiers)	[20]
Double spiral	Dean flow	MCF-7, Hela in whole blood	W = 300 H = 50	3.33 × 10^7^ cells/min	88.5%	[22]
Slanted spiral	Dean flow	T24, MCF-7, MDA-MB-231 in blood	W = 600; H = 80 /130 (inner/outer)	1700 μL/min.	η = 80% (T24) 85% (MCF-7) 87% (MDA-MB-231).	[23]
Symmetrical serpentine	Inertial	polystyrene beads (D = 3, 10; 5, 13)	W = 200 H = 42	600 μL/min	>97% (large particles); >92% (small particles)	[31]
Serpentine with Suction channel	Inertial, Dean flow and suction	RBC, WBC (D = 8, 15).	W = 200 H = 58	1 × 10^8^ cells/sec, or 250 μL/sec	93.2%	Our Work

## References

[1] Anker P., Mulcahy H., Chen X.Q., Stroun M. (1999). Detection of circulating tumour DNA in the blood (plasma/serum) of cancer patients. Cancer Metastasis Rev..

[2] Bhagat A.A., Kuntaegowdanahalli S.S., Papautsky I. (2008). Continuous particle separation in spiral microchannels using Dean flows and differential migration. Lab Chip.

[3] Hou H.W., Bhagat A.A.S., Lee W.C., Huang S., Han J., Lim C.T. (2011). Microfluidic Devices for Blood Fractionation. Micromachines.

[4] Catarino S.O., Rodrigues R.O., Pinho D., Miranda J.M., Minas G., Lima R. (2019). Blood cells separation and sorting techniques of passive microfluidic devices: From fabrication to applications. Micromachines.

[5] Lin C.-H., Lee C.-Y., Tsai C.-H., Fu L.-M. (2009). Novel continuous particle sorting in microfluidic chip utilizing cascaded squeeze effect. Microfluid. Nanofluidics.

[6] Yamada M., Seki M. (2005). Hydrodynamic filtration for on-chip particle concentration and classification utilizing microfluidics. Lab Chip.

[7] Morijiri T., Sunahiro S., Senaha M., Yamada M., Seki M. (2011). Sedimentation pinched-flow fractionation for size- and density-based particle sorting in microchannels. Microfluid. Nanofluidics.

[8] Lin B.K., McFaul S.M., Jin C., Black P.C., Ma H.S. (2013). Highly selective biomechanical separation of cancer cells from leukocytes using microfluidic ratchets and hydrodynamic concentrator. Biomicrofluidics.

[9] Takagi J., Yamada M., Yasuda M., Seki M. (2005). Continuous particle separation in a microchannel having asymmetrically arranged multiple branches. Lab Chip.

[10] Chen X., Cui D., Liu C., Li H. (2008). Microfluidic chip for blood cell separation and collection based on crossflow filtration. Sens. Actuators B Chem..

[11] Sollier E., Rostaing H., Pouteau P., Fouillet Y., Achard J.-L. (2009). Passive microfluidic devices for plasma extraction from whole human blood. Sens. Actuators B Chem..

[12] Alvankarian J., Bahadorimehr A., Majlis B.Y. (2013). A pillar-based microfilter for isolation of white blood cells on elastomeric substrate. Biomicrofluidics.

[13] Li X., Chen W., Liu G., Lu W., Fu J. (2014). Continuous-flow microfluidic blood cell sorting for unprocessed whole blood using surface-micromachined microfiltration membranes. Lab Chip.

[14] Chiu Y.-Y., Huang C.-K., Lu Y.-W. (2016). Enhancement of microfluidic particle separation using cross-flow filters with hydrodynamic focusing. Biomicrofluidics.

[15] Chen C.L., Chen K.C., Pan Y.C., Lee T.P., Hsiung L.C., Lin C.M., Wo A.M. (2011). Separation and detection of rare cells in a microfluidic disk via negative selection. Lab Chip.

[16] Hur S.C., Mach A.J., di Carlo D. (2011). High-throughput size-based rare cell enrichment using microscale vortices. Biomicrofluidics.

[17] Zhou J., Kasper S., Papautsky I. (2013). Enhanced size-dependent trapping of particles using microvortices. Microfluid. Nanofluidics.

[18] Hahn Y., Hong D., Kang J., Choi S. (2016). A reconfigurable microfluidics platform for microparticle separation and fluid mixing. Micromachines.

[19] Choi S., Park J.-K. (2007). Continuous hydrophoretic separation and sizing of microparticles using slanted obstacles in a microchannel. Lab Chip.

[20] Di Carlo D., Edd J.F., Irimia D., Tompkins R.G., Toner M. (2008). Equilibrium separation and filtration of particles using differential inertial focusing. Anal. Chem..

[21] Ramachandraiah H., Ardabili S., Faridi A.M., Gantelius J., Kowalewski J.M., Mårtensson G., Russom A. (2014). Dean flow-coupled inertial focusing in curved channels. Biomicrofluidics.

[22] Sun J., Li M., Liu C., Zhang Y., Liu D., Liu W., Jiang X. (2012). Double spiral microchannel for label-free tumor cell separation and enrichment. Lab Chip.

[23] Warkiani M.E., Guan G., Luan K.B., Lee W.C., Bhagat A.A.S., Chaudhuri P.K., Lim C.T. (2014). Slanted spiral microfluidics for the ultra-fast, label-free isolation of circulating tumor cells. Lab Chip.

[24] Kim T.H., Yoon H.J., Stella P., Nagrath S. (2014). Cascaded spiral microfluidic device for deterministic and high purity continuous separation of circulating tumor cells. Biomicrofluidics.

[25] Burke J.M., Zubajlo R.E., Smela E., White I.M. (2014). High-throughput particle separation and concentration using spiral inertial filtration. Biomicrofluidics.

[26] Vasseur P., Cox R.G. (2006). The lateral migration of spherical particles sedimenting in a stagnant bounded fluid. J. Fluid Mech..

[27] Yang S., Lee S.S., Ahn S.W., Kang K., Shim W., Lee G., Kim J.M. (2012). Deformability-selective particle entrainment and separation in a rectangular microchannel using medium viscoelasticity. Soft Matter.

[28] Chung A.J. (2019). A Minireview on inertial microfluidics fundamentals: Inertial particle focusing and secondary flow. BioChip J..

[29] Zhou J., Papautsky I. (2013). Fundamentals of inertial focusing in microchannels. Lab Chip.

[30] Chen Y.-L. (2014). Inertia- and deformation-driven migration of a soft particle in confined shear and Poiseuille flow. RSC Adv..

[31] Zhang J., Yan S., Sluyter R., Li W., Alici G., Nguyen N.T. (2014). Inertial particle separation by differential equilibrium positions in a symmetrical serpentine micro-channel. Sci. Rep..

[32] Yoon D.H., Ha J.B., Bahk Y.K., Arakawa T., Shoji S., Go J.S. (2009). Size-selective separation of micro beads by utilizing secondary flow in a curved rectangular microchannel. Lab Chip.

[33] Cruz J., Graells T., Walldén M., Hjort K. (2019). Inertial focusing with sub-micron resolution for separation of bacteria. Lab Chip.

[34] Amini H., Sollier E., Weaver W.M., di Carlo D. (2012). Intrinsic particle-induced lateral transport in microchannels. Proc. Natl. Acad. Sci. USA.

[35] Liu C., Hu G. (2017). High-throughput particle manipulation based on hydrodynamic effects in microchannels. Micromachines.

[36] Zhang J., Li W., Alici G. (2017). Inertial microfluidics: Mechanisms and applications. Advanced Mechatronics and MEMS Devices II.

[37] Lee D.J., Brenner H., Youn J.R., Song Y.S. (2013). Multiplex particle focusing via hydrodynamic force in viscoelastic fluids. Sci. Rep..

